# Agency Staff Utilization in Nursing Homes and Assisted Livings: Patterns and Concerns

**DOI:** 10.1093/geroni/igaf122.2684

**Published:** 2025-12-31

**Authors:** Patrick Mese, Kennedy Berner, John Bowblis

**Affiliations:** Miami University, Oxford, Ohio, United States; Miami University, Oxford, Ohio, United States; Miami University, Oxford, Ohio, United States

## Abstract

Since 2020, long-term care providers have been faced with a shortage of direct care workers. As a result, many nursing homes (NH) and assisted livings (AL) have turned to staffing agencies. Agency staffing is believed to be associated with worse resident outcomes, but our understanding of how and why providers use agency staff, and the implications of their use is limited, especially in ALs. Using data from the 2023 Ohio Biennial Survey (> 90% response rate), NHs and ALs that utilized agency staff for nursing aides or personal care aides during the year were identified. Nearly 68% of NHs and 38% of ALs utilized agency staff. NHs and ALs are more likely to use agency staff for hard-to-fill weekend and overnight shifts (NHs: 77%, ALs: 60%), filling in for last-minute call-offs (78%, 67%), when an employee leaves employment (76%, 64%) or is vacationing (55%, 44%), and when there is an increase in occupancy (59%, 27%). Over 55% of NHs and 39% of ALs reported using agency staff as a substitute for a directly employed aide for over 4 weeks. The most common serious concern among administrators regarding agency staff are costs, lack of dedication to building, negatively impacting the morale of directly employed staff, inadequate time to know residents’ care plans, and lack of knowledge of building policies and procedures. Investment in the long-term care workforce is necessary to reduce the use of agency staff. This study provides important insights that help direct these investments.

